# The NOMAD mini-apps: A suite of kernels from ab initio electronic structure codes enabling co-design in high-performance computing

**DOI:** 10.12688/openreseurope.16920.3

**Published:** 2025-04-10

**Authors:** Isidre Mas Magre, Rogeli Grima Torres, José María Cela Espín, José Julio Gutierrez Moreno

**Affiliations:** 1 Barcelona Supercomputing Center (BSC), Plaça Eusebi Güell, 1-3, Barcelona, 08034, Spain

**Keywords:** High-performance computing, exascale, ab initio calculations, materials science, eigensolver library, Green's function methods, co-design, mini-apps

## Abstract

This article introduces a suite of mini-applications (mini-apps) designed to optimise computational kernels in *ab initio* electronic structure codes. The suite is developed from flagship applications participating in the NOMAD Center of Excellence, such as the ELPA eigensolver library and the *GW* implementations of the exciting, Abinit, and FHI-aims codes. The mini-apps were identified by targeting functions that significantly contribute to the total execution time in the parent applications. This strategic selection allows for concentrated optimisation efforts. The suite is designed for easy deployment on various High-Performance Computing (HPC) systems, supported by an integrated CMake build system for straightforward compilation and execution. The aim is to harness the capabilities of emerging (post)exascale systems, which necessitate concurrent hardware and software development — a concept known as co-design. The mini-app suite serves as a tool for profiling and benchmarking, providing insights that can guide both software optimisation and hardware design. Ultimately, these developments will enable more accurate and efficient simulations of novel materials, leveraging the full potential of exascale computing in material science research.

## Introduction

Exascale computing represents a significant advancement in High-Performance Computing (HPC), unlocking unprecedented opportunities to transform computational modelling. In this context, *ab initio*-based materials modelling codes are in the ideal situation to take advantage of this revolution, which will provide all the resources to enable more accurate calculations, larger size scales and high-throughput exploration of data sets for the discovery of novel materials ^1^. However, the distinguishing features of these new supercomputers include increased heterogeneity in their architectures, often incorporating specialised accelerators designed for specific applications ^2^. To fully realise the potential of the exascale era, developers of *ab initio* electronic structure codes are investing efforts in exploiting the advanced capabilities of the new supercomputers ^3^. Some efforts include the development of low-scaling algorithms for existing methods as well as developing efficient shared libraries for the most critical and computationally expensive parts of the codes. Some examples of popular libraries are; ELPA ^4^, an efficient eigensolver for petaflop (and exascale) systems, libxc ^5^, a library of exchange and correlation functionals, GreenX ^6^, an open-source library that supports exascale implementations of Green’s-function-based methodologies, SIRIUS ^7^, a domain-specific library for electronic structure calculations, ELSI ^8^, an open infrastructure for electronic structure solvers, and PEXSI ^9, 10^, a fast method for electronic structure calculation based on Kohn-Sham density functional theory (DFT). Many efforts are also focused on the development of workflow managers and job schedulers for high-throughput computations (HTC) ^11^, some examples are AiiDA ^12^, the Atomic Simulation Environment (ASE) ^13^ and Atomic Simulation Recipes (ASR) ^14^, or FireWorks ^15^. With all these active developments in software and hardware, co-design is also an important effort that is bound to play a crucial role in an efficient transition to the (post)exascale era.

**Figure 1.**
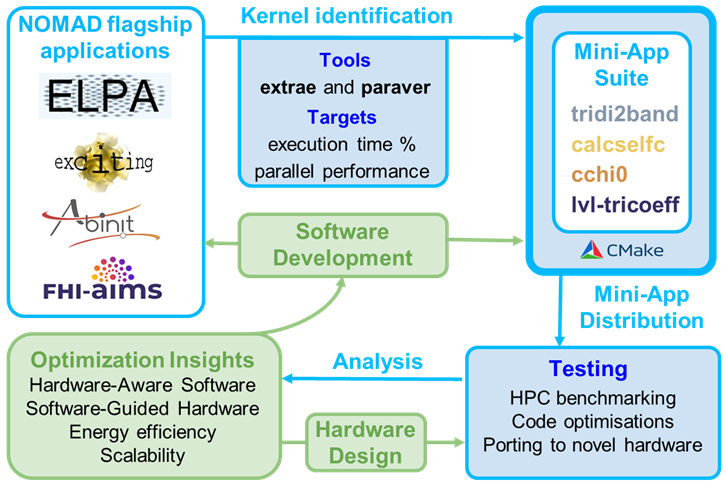
Overview of the co-design workflow for NOMAD CoE flagship applications. The top panels show the one-to-one correspondence between codes and kernels extracted for the mini-apps. Performance analysis tools and metrics are also indicated. Examples of current usage examples of the suite are displayed in the testing section. Possible optimisation targets are displayed in the green panels.

Co-design is intended to facilitate the paradigm shift by concurrently and cooperatively developing both, hardware and software ^16^. There are some tangible examples of co-design efforts focused on atomic-scale simulations. For example, while the Gromacs molecular dynamics (MD) code was ported to GPUs, NVIDIA also introduced stream priority bits in their hardware, which eventually benefited the communications and led to better code performance ^17^. Another interesting example is the Anton supercomputer, which was originally designed to efficiently run classical MD simulations ^18^. For this purpose, and being one of the most relevant applications and family of applications with more users in the HPC community, the performance of *ab initio* electronic structure codes has to be systematically profiled on new infrastructures. Therefore, effective communication between hardware engineers, software developers and users is especially critical during the systems’ design process. However, the extreme complexity of these codes, being many of them collaboratively developed among large research teams with rotating staff and for many decades, makes these tasks quite challenging. Therefore, it is always important to set a visible milestone in the current software status and try to achieve the optimum performance of novel HPC features at the earliest possible stage, when low-level (i.e. bit-level or compiler) adaptations are still possible. Otherwise, benchmarking would only be possible once the development of the system approaches production capabilities. This problem can primarily be addressed using simplified models, such as mini-apps, which are proxies of full code executions ^19^. An overview of the co-design workflow and the mini-app suite presented in this paper is shown in Figure 1.

Mini-apps strive for a simple compilation and small computing times, enabling a rapid iteration of code and leaving room for low-level improvements in hardware at experimental platforms. A mini-app comprises a small fraction of the code length and complexity with respect to its parent application while it retains the primary performance-intensive aspects. Therefore, mini-app benchmarking can inform the implementation of new HPC systems more effectively. Ideally, this would be a continuous process throughout the implementation and in the initial design phase of the systems. Mini-apps have the potential to support our efforts in co-design as the performance metrics will be easily transferable between selected HPC systems and pre/post-exascale prototypes more effectively. Moreover, mini-apps can also serve to inform developers of the parent applications of the feasibility of potential directions for future developments. An illustrative example of a mini-app suite is the one developed by the *arch* project, which unified several physical simulations such as heat transfer, gravity or hydrodynamics problems within a consistent coding practice under a common infrastructural layer ^20^. Also, contributors to the *Mantevo* project have focused on developing tools to accelerate and improve the HPC by providing application and library proxies. This suite includes some applications that could be useful for materials research, such as classical MD or finite elements mini-apps ^21^. Also, a methodology paper for showing the link between full application codes and their proxies was published ^22^. In addition to all these, and following a similar spirit, the Sustained System Performance (SSP) (and its simplified (SSSP) metric) enable performance projection that correlates with full applications from a suite of mini-apps, providing a more direct estimation of the application performance in contrast to for example the popular Linpack benchmark ^23^.

On this basis, our article presents a suite of mini-apps developed from a set of representative *ab initio* electronic structure codes that are part of the Novel Materials Discovery (NOMAD) Centre of Excellence (CoE) ^24^, all using different basis sets and aiming to be a seed of collaboration in the co-design endeavour. The objective of the project is to facilitate systematic studies and predictions of novel materials enabled by upcoming exascale computing. In the following sections, first we introduce the importance of eigensolvers in electronic structure calculations and sketches the *GW* approximation to serve as context and reference to present the kernels identified for each mini-app. The next section describes the methods used to profile the codes, identify the kernels and develop the mini-apps suite. Then we proceed to describe the suite and how to operate the mini-apps within the suite. In the end, we give some conclusions and motivate further research that could be carried out using the NOMAD mini-apps.

## Theoretical background

The mini-apps currently present in the suite correspond to isolated kernels both from the ELPA library (version 2022.05.001), an efficient eigenvalue solver for HPC applications ^4, 25^, and from the *GW* implementation of the *ab initio* codes exciting (oxygen version) ^26, 27^, which uses all-electrons with linearised augmented plane-wave plus local orbitals (LAPW+lo), Abinit (version 9.6.2) ^28– 30^, which uses plane waves and pseudo-potentials (PW+PP), and FHI-aims (version 210716_1) ^31, 32^, which uses all-electrons with numeric atom-centered orbitals (NAOs).

### Eigenvalue problems

Eigenvalue problems are common tasks in ab initio electronic structure calculations when solving the Schrödinger equation or approximations of it, such as those found in Density Functional Theory (DFT) and many-body perturbation theories (MBPT) like the *GW* approximation. Eigensolvers are often the main computational bottleneck in Density Functional calculations, expending in large cases the vast majority of the total computational cost and, in practice, also limiting the systems’ sizes. For researchers in the field of computational materials science, an efficient and scalable solution to the eigenvalue problem is thus of major importance. The ELPA library ( *E*igenvalue *S*o *L*vers for *P*etaflop- *A*pplications) is designed for exascale HPC. The ELPA scalability and parallelisation capabilities make it a key tool for handling computationally intensive tasks in material discovery and design ^4^. ELPA is a well-established solver library, and today, it has interoperability with the majority of the most widely used *ab initio* packages, making it an indispensable component in the computational toolkit for advancing material science research.

### The *GW* approximation

The *GW* approximation of Hedin’s equations represents a significant advancement in accuracy for electronic structure calculations, for example, enhancing the prediction of band gaps in semiconducting materials ^33^. This improvement is notable when compared to traditional Density Functional Theory (DFT) with Local Density Approximation (LDA) or General Gradient Approximation (GGA) functionals. A key element of this advancement is the introduction of the self-energy, Σ, in Hedin’s equations, which encapsulates all electron-electron interactions extending beyond the Hartree energy. This incorporation addresses the limitations in conventional DFT methods, which often underestimate electron correlation effects crucial for accurate band gap predictions.

In practical implementations, especially where computational efficiency is paramount, the self-consistent *GW* formalism often gives way to a simplified approach termed single-shot *GW* or *G*_0_ *W*_0_. This approximation involves using the non-interacting Green’s function, *G*_0_, in place of the fully interacting *G*. The equation for *G*_0_ is given by:


$$G_{0}\left(r,r^{\prime };\omega \right)=\sum_{nk}\frac{\psi_{nk}\left(r\right)\psi_{nk}^{*}\left(r^{\prime }\right)}{\omega –\varepsilon_{nk}–i\eta }\;(1)$$

Here, ψ *_nk_* ( ***r***) and ε *_nk_* represent the DFT eigenfunctions and eigenvalues, respectively, with *n* and *k* denoting band and k-point indices in the Brillouin zone and *ω* is the frequency of the Green’s function. The term *η* is a small, positive (negative) number for occupied (unoccupied) states, ensuring numerical stability.

Similarly, the screened Coulomb interaction *W* in the *GW* formalism is approximated by *W*_0_, described as:


$$W_{0}\left(r,r^{\prime };\omega \right)=\;\int v\left(r_{1},r^{\prime }\right)\varepsilon^{–1}\left(r,r_{1};\omega \right)dr_{1}\;(2)$$

In this context, *ν*( ***r***, ***r′***) denotes the bare Coulomb interaction, and *ϵ* ^−1^( ***r***, ***r***_1_; *ω*) is the inverse dielectric function, reflecting the material’s response to electronic perturbations.

More detailed descriptions of the theory and applications of the *GW* method can be found in more specific publications ^34, 35^.

## Methods

The identification of the relevant kernels on the exciting, Abinit, FHI-aims codes and the ELPA library was done by executing and profiling benchmarks in the MareNostrum-4 supercomputer. For this we used a set of profiling tools:

- Extrae ^36^ is a tracing tool. It collects information such as execution times, MPI and OpenMP calls, and performance counter information with a Performance Application Programming Interface (PAPI) ^37^, supplying a consistent interface and methodology for collecting performance counter information from various hardware and software components.
- Paraver ^38, 39^ takes traces generated with Extrae and provides a visual interface to analyse them. Trace figures in the manuscript were produced with this software.

These tools identified relevant kernels by comparing execution times and performance metrics related to their parallel performance (load imbalance, parallel efficiency, etc.). The compute-intensive regions of the code were found to be associated with the relevant computations in the implementations above. We have performed exhaustive performance analyses using different test cases for each of the applications and performed scaling tests on the number of MPI/OMP processes to accommodate the available memory resources within a reasonable time to solution. We selected at least two realistic test cases for all the applications with increasing complexity computational cost. In ELPA, we used matrices of different sizes. For the *GW* applications, we selected ZrO _2_ as a common test case for all the codes, which is a technologically relevant semiconductor with applications in catalytic green energy production. The alternative test cases were differently adapted for each code and described in their corresponding sections. Once kernels in the original source code are identified, the mini-apps are developed through the migration of these to a stand-alone implementation. Data dependencies are addressed through the insertion of a checkpoint right before the kernel in the original execution, which captures all the relevant variables and parameters and serves as the input file for the mini-apps. To avoid extra dependencies, such as HDF5 or other common checkpointing modules, the checkpoints are implemented through a binary I/O wrapper included in the mini-apps distribution.

The mini-apps are integrated into a CMake build system, ensuring ease of compilation across a variety of architectures in HPC environments. All mini-apps are written in FORTRAN, the language that is mostly used in their parent applications. Our testing has covered a range of machines, including those with Intel, POWER9, and AMD processors. Given the diversity of these architectures, it is certainly worthwhile to maintain this broad testing approach. The full suite of mini-apps is compiled simultaneously, streamlining the setup process as the environment remains consistent for all mini-apps during testing. This integrated build system not only simplifies the initial setup but also enhances the suite’s extensibility for incorporating new mini-apps or updating existing ones.

## Mini-apps suite

The Mini-apps Suite consists of four mini-apps. Each mini-app is described below by naming the source file and subroutines containing the selected kernel and a justification for its selection. The kernels might have dependencies to other code regions; those are assumed to be included in the mini-app as well, but special care was taken not to include dependencies that are not strictly required and to reduce the mini-app source code to the minimum.

### ELPA mini-app

The ELPA mini-app isolates the *trans_ev_tridi_to_band* subroutine, located in the source code *elpa2_trans_ev_tridi_to_band_template.F90*. The ELPA library implements different methods to solve the eigenvalue problem; among those methods, we selected the two-stage tridiagonalisation, which includes an intermediate reduction to bidiagonal form before doing a reduction to tridiagonal form, as opposed to the one-stage method, which reduces the original matrix directly to tridiagonal form ^25^. The two-stage method is normally preferred to the one-stage one in large problems and when most of the eigenvectors need to be computed. Other choices made in the method selection are the type and precision of the numbers. Our mini-app uses real numbers with double precision and employs a generated random matrix as input.

**Figure 2.**
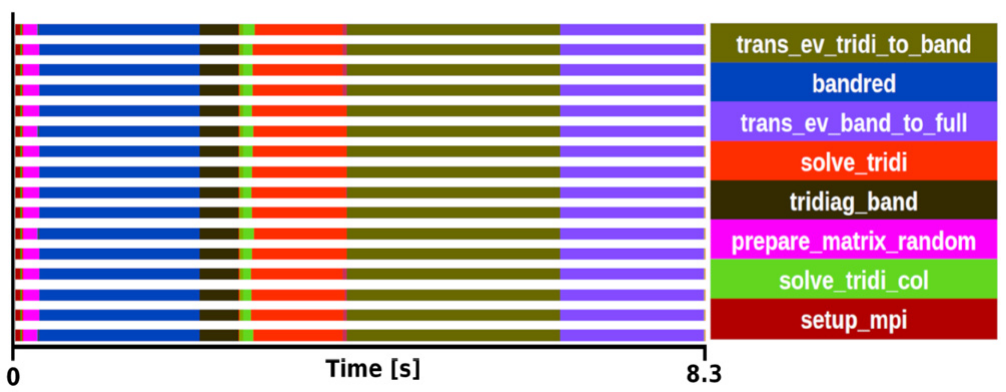
Trace of ELPA run on a matrix size of 8000 × 8000 with the AVX512 kernel. The runtime was 8.3 s in 16 one-threaded MPI processes.

The kernel selection was done after profiling two different executions. The first was a medium-sized problem with a matrix size of 8000 *×* 8000 executed with a block size of 16 on 16 processors with an execution time of 8.3 s (see Figure 2), the second was a large matrix of size 100000 *×* 100000 executed with a block size of 24 on 192 processors with an execution time of 969 s. Both traces showed similar behaviour in terms of task distribution and relative weight of the routines in the trace.

After analysing the several steps to compute the two-stage method, we concluded that the most suitable function for the mini-app is *trans_ev_tridi_to_band*. There are some good reasons for this selection. First, this is the most computationally expensive step in both experiments, becoming more important as the system size increasing. In particular, our perfornace analyses show that *trans_ev_tridi_to_band* takes 32% of the total time in the smaller test case, and 39% in the large one. Also, it does not depend on external functions, while other functions heavily rely on external libraries such as BLAS, ESSL or KML. The function is not dominated by DGEMM or communications. It is also relevant to note that the developers have put substantial efforts into improving this subroutine on multiple architectures. In the hypotetical case of not having this routine optimally vectorized for a given architecture this, kernel would become largely predominant over other subroutines. Therefore, its relative weight makes it a good target to test on experimental hardware where vectorization might not be supported yet. Today, it supports SSE, AVX(2/512), SPARC64 SSE, ARM SVE(128/256/512), BlueGene/(P/Q), NVIDIA, AMD and Intel GPUs.

### exciting mini-app

**Figure 3.**
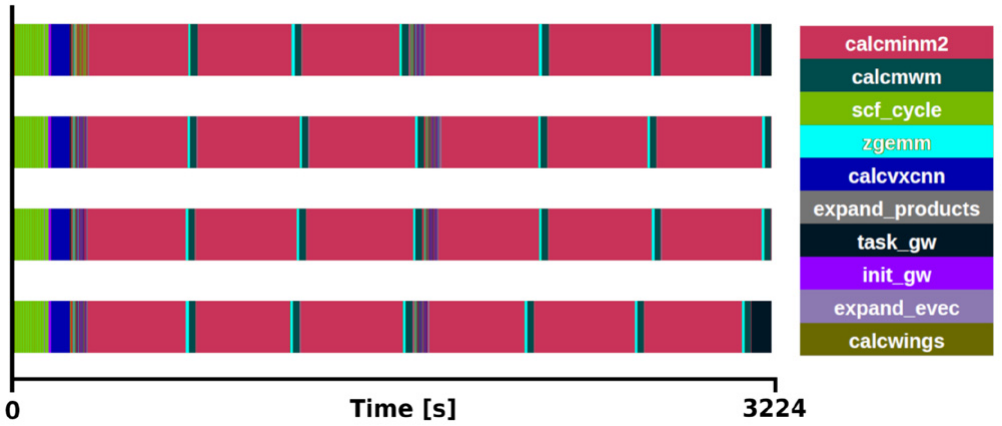
Trace of exciting full execution. ZrO _2_ primitive cell (3 atoms) with 2×2×2 q/k-point grid and 800 bands. The runtime was 3214 s with 4 MPI processes and 48 threads each.

**Figure 4.**
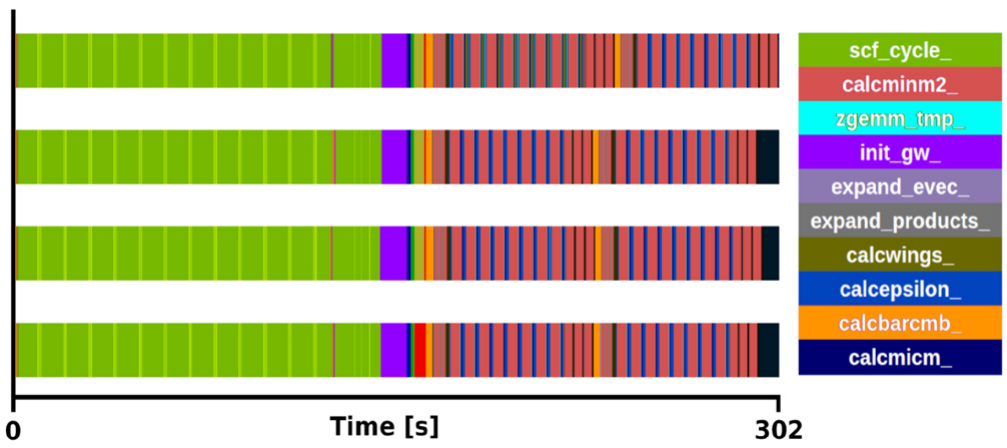
Trace of exciting full execution. ZrO _2_ primitive cell (3 atoms) with 2×2×2 q/k-point grid and 12 bands. The runtime was 302 s with 4 MPI processes and 48 threads each.

The exciting mini-app isolates the subroutines *expand_products* and *calcmwm* called from the source code *calcselfc.f90*. This source code is called in the last step for the calculation of the self-energy in the main loop of the Brillouin zone’s integration ^26^. The selection of the kernel was done after profiling different executions of the benchmark ZrO _2_ primitive cell (3 atoms) with a 2 *×* 2 *×* 2 q/k-point grid, one with all (800) bands and another with a reduced number (12) of bands, see Figure 3 and Figure 4 for the respective traces.

The benchmarks were executed using 4 MPI processes with 48 OpenMP threads each. It was observed that the main loop for the Brillouin Zone was distributed in 8 tasks (1 task per k-point) among the available MPI processes. The execution time of the *G*_0_ *W*_0_ implementation of exciting is therefore explained by the duration of one of these tasks. Each of these tasks is composed of the computations of the necessary quantities in the *G*_0_ *W*_0_ formalism using an auxiliary mixed product basis. In all these computations, a product expansion is needed to perform the calculations, and therefore, the *expand_products* subroutine is a common call in all the calculations. Here we should remind that as a beyond DFT method, the *G*_0_ *W*_0_ requires a previous calculation of the density matrix at the ground state ( *scf_cycle*) which is not considered for the development of the exciting, Abinit, and FHI-aims mini-apps. The amount of calls to *expand_products* and its execution time depends on the subroutine and the system’s size. For the benchmark, it was decided to focus only on the duration and performance of the *GW* implementation, not taking into account the ground state section. When all the bands are used, the subroutine *calcminm2* (magenta sections in Figure 3) called within *expand_products*, used for the calculation of the self-energy, is the most time-consuming section, increasing the total runtime to 3214 s. This subroutine alone takes 85% of the *G*_0_ *W*_0_ task. Moreover, considering that *calcmwm* takes another 5% of the *G*_0_ *W*_0_ task, the exciting mini-app presented here represents 90% of the *G*_0_ *W*_0_ implementation for this specific benchmark. The other 10% is devoted to the calculation of other *G*_0_ *W*_0_ quantities, which also execute the subroutine *expand_products* and are expected to become proportionally more relevant depending on the number of bands used in the calculation. In the reduced case ( Figure 4), the routines included in the mini-app ( *expand_products and calcwm*) still consume a 58% of the *G*_0_ *W*_0_ task execution time.

### Abinit mini-app

**Figure 5.**
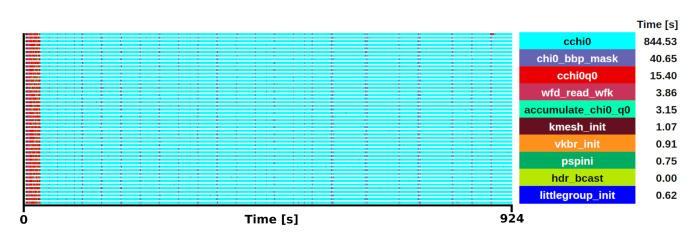
Trace of Abinit execution of the screening calculations for 35 q-points in a ZrO _2_ system. The runtime was 924 s in 48 one-threaded MPI processes.

The Abinit mini-app isolates one iteration of the loop over q-points for the polarizability *χ*_0_ calculation in the screening step. This is a triple loop that iterates over k-points, conduction, and valence bands, these quantities are nested forming the triple loop ^29^. This kernel is located in the *m_chi0.F90* source code inside the *cchi0* subroutine which is called for every q-point different from the Γ-point. The benchmark was executed in 48 one-threaded MPI processes and the runtime was 924 s for the screening step, from which 88% executes the subroutine *cchi0* over 35 q-point iterations. Figure 5 shows the trace of this execution.

The mini-app includes the initialisation of the *bbp_ks_distrb* matrix, which reduces the size of the checkpoint by around two orders of magnitude, from GB to a few MB. Then, it executes a loop over k-points and bands to calculate the *chi0* matrix. Within this loop, two subroutines were identified as the main computational kernels, namely *rho_tw_g* and *assemblychi0_sym*, which together use 70% of the execution time for *cchi0*, while the remaining 30% are memory accesses and variable updates for each iteration in the nested loops. The selection of the kernel was done after profiling the different steps involved in the *GW* calculations for a small 3-atom ZrO _2_ and a large 11-atom Zr _2_Y _2_O _7_ primitive cell system, from which it was observed that the calculation of the screening *W*_0_ is the most time-consuming for the *G*_0_ *W*_0_ implementation.

In this case, the extraction of only one of these q-point iterations and the portion executed in a single thread is enough to represent the full code execution, as there are no MPI communications within the selected kernel. With this selection of kernel, the mini-app reduces an execution of 15 minutes running in 48 MPI processes to 14 seconds in a single process, while it still represents the 88% of the code execution time. For simplicity, the Zr _2_Y _2_O _7_ the calculation was done at 3 q-points, being one of the Γ-point. In this reduced case, the chi0 routine takes a 42% of the computational time, however, if we extrapolate this to a potentially extended calculation using a 36 q-point grid, we expect the routines in the mini-app to represent a 92% of the total execution.

### FHI-aims mini-app

**Figure 6.**
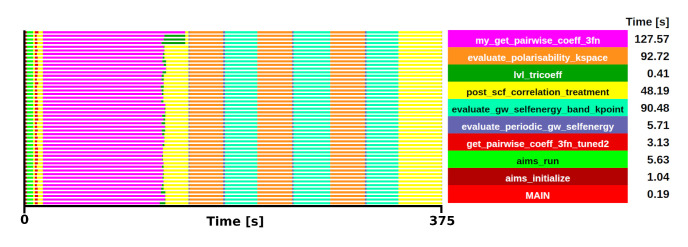
Trace of FHI-aims execution of a Si system. The runtime was 375 s in 48 one-threaded MPI processes.

The FHI-aims mini-app isolates the routine that computes the LVL triple expansion coefficients in real space, and Fourier-transforms the LVL triple coefficients, which is called *gw_init_lvl_tricoeff_recip*. These coefficients expand the product of wavefunctions to the so-called Auxiliarity Basis Functions (ABF). These ABF are atomically-centred, and they are constructed similarly to the mixed product basis in the LAPW framework but without the plane-wave component in the interstitial region. This basis set expansion is used to achieve efficient implementations of Hartree-Fock, second-order Moller-Plesset perturbation theory (MP2), the Random Phase Approximation (RPA), and *GW* within the numerically tabulated atom-centered orbitals (NAO) basis set framework ^32, 40^. This renders the mini-app as a relevant kernel for all these methods. The FHI-aims mini-app tested a code version that is no longer current and has since been optimised, particularly in the LVL part. While this older version of the code is not distributed anymore, the mini-app can still be useful to benchmark different hardware platforms in a practical computational case. The selection of the kernel was done after profiling a simple Si system in 48 MPI processes and a more complex ZrO _2_ system in up to 384 MPI processes. Checkpoint inputs for both cases have been included in the repository. For simplicity of visualisation, the trace of the Si benchmark is shown in Figure 6.

From the trace, two initial candidates were identified as potential kernels for the mini-app, with comparable execution times: *gw_init_lvl_tricoeff_recip*, which includes the subroutine *my_get_pairwise_coeff_3fn* (shown in magenta Figure 6) and *evaluate_periodic_gw_selfenergy*, which includes the subroutines *evaluate_polarisability_kspace* and *evaluate_gw_selfenergy_band_kpoint* (shown in orange and cyan respectively). Among these, the selection of *gw_init_lvl_tricoeff_recip* was made due to the limited influence of MPI communications (compared to *evaluate_periodic_gw_selfenergy*) and its shared applicability with several beyond-DFT methods. The behaviour and relative weight is consistent in both test cases. The selected kernel in the Si system has an execution time of 127.57 s, which is a 34% of the total execution, while in the ZrO _2_ system, the execution time of the kernel was 978.53 s, accounting for a 45% of the total execution.

## Operation

The Mini-App Suite is intended to be easily deployed and executed on different machines. To facilitate this, the suite has an integrated compilation and execution scheme for all the mini-apps using a CMake build system. Instructions to build, compile and execute the suite are provided in more detail within the README file included in root of the repository.

Once the suite has been properly built, the checkpoint files (stored in Zenodo ^41^) must be downloaded and copied into the benchmark folder found in the repository. This can be directly done by executing the bash script *get-ckpts.sh* inside the benchmarks folder. However, in any case, downloading of the checkpoints should happen automatically when attempting to execute any of the run scripts provided, which are designed to check for the presence of the checkpoints in the expected locations and download them in case they are not found. In case the machine has no internet access, the file can be downloaded following the link provided in the README file. If a user would like to use alternative material benchmark systems, these can be generated by copying the checkpoint generator files placed inside the repository folder *utils* onto the original code, recompiling it and executing the modified version of the code using the desired test case and input parameters. This will generate a new checkpoint file that can be loaded within the mini-apps.

Examples of execution scripts are provided with the benchmark of each mini-app. The corresponding binaries for the *GW* cases must be executed, providing a checkpoint file. The mini-app should be submitted to a queuing system; for that, submission (SLURM) scripts used in MareNostrum-5, MareNostrum-4, and CTE-POWER (BSC, Spain), Leonardo (CINECA, Italy), LUMI (CSC, Finland), Vega (IZUM, Slovenia) and Karolina (IT4I, Czech Republic) are provided. Benchmark scripts launch multiple executions, increasing the number of processes until they fill all the CPUs of the machine. Users should fill a configuration file with some of the specifications of the machine, the available toolchains and specific flags for their queue system. After their execution, the mini-apps will produce a summary report with information on performance metrics such as timings and numerical checks. The information produced from the reports can be readily used to get insights into the performance. The user can, for instance, compare how the different mini-apps perform on different machines, analyse the potential degradation of the single-core performance with the occupancy of the node, or test different compiling options. More detailed performance analyses of the mini-apps for specific systems are left to be done by experienced users.

## Conclusions

The NOMAD mini-app suite, presented in this article, includes four mini-apps extracted from a set of representative flagship codes within the *ab initio* electronic structure community. These mini-apps focus on critical computational kernels involved in the *GW* implementations of exciting, Abinit and FHI-aims, and the ELPA eigenvalue solver. This mini-app suite represents a pragmatic approach to facilitate co-design efforts, employing *ab initio* electronic structure applications as use cases. By isolating and targeting specific computational kernels, this suite not only offers a pathway for focused optimisation but also marks a significant stride in fine-tuning both software and hardware capabilities in tandem. This aspect is particularly relevant as we venture into the era of exascale computing.

The practical benefits of these mini-apps are further enhanced by their adaptability across various HPC platforms. Their user-friendly deployment, facilitated by a streamlined CMake build system, broadens their accessibility to a diverse group of researchers and developers. This accessibility is pivotal in fostering a collaborative environment, essential for the co-evolution of computational tools and hardware technologies. The NOMAD suite are being used to continuously benchmark the supercomputers that are currently being deployed by the European High Performance Computing Joint Undertaking (EuroHPC JU), all having different hardware architectures and compilers/software stacks. These outcomes of these activities provide useful feedback to integrators, system administrators and users. In addition, the ELPA mini-app being used as a case study for the development of novel hardware prototypes, which are currently being designed to be used in future (post-)exascale platforms. These experimental architectures perform normally on emulators or Field Programable Gate Array (FPGA) based single-node platforms with reduced clock time and limited availability in terms of compiler stacks and libraries. Therefore, the initial porting activities of complex codes to these frameworks is only feasible at the mini-app level. This is a clear example of co-design, in which the software is adapted to perform efficiently in these novel architectures, while we are providing constant feedback to the hardware architects and compiler developers at the early stage of development ^42^.

While these mini-apps serve as a valuable asset in electronic structure calculations, it is essential to recognise that their role is one piece of a giant puzzle. As we continue to explore the vast potential of exascale computing, it is crucial to maintain an ongoing dialogue within the community, ensuring that these tools evolve in response to emerging challenges and opportunities.

We should also recall that while DFT has been so far the main workhorse of the ab initio electronic structure community, its accuracy estimating properties such as electronic band gaps may need to be improved for some applications. Therefore, the use of more accurate (and computationally expensive) beyond-DFT methods such as GW is required. We strongly believe that with the increasing computational power that will come along with the arrival of the (post)exascale era, these methods will increasingly become more accessible and popular among the community, and this is the main reason that drove us to choose GW for the majority of the selection of these mini-apps. It is also important to point out that in addition to the current predominance of GPUs, the future of HPC will also increase in hardware heterogeneity (some of them based on CPUs, which could operate larger vectors, e.g. VPUs) and new programming models. Our mini-apps could also be helpful as a starting point for implementing new porting strategies and optimisations before merging them with the complete code.

The mini-apps suite, the codes that make part of it, and, of course, the whole HPC ecosystem are quite lively research fields and in constant development. Therefore, performance metrics should be properly documented and shared among the community to facilitate the co-design efforts. Our repository is open to incorporating new performance metrics when the mini-apps are executed on new machines. Contributions of new versions that attempt to optimise the existing kernels or new mini-apps addressing other kernels are also expected as the co-design activity develops.

## Software availability

The initial release of the mini-apps suite, including the exciting, Abinit and ELPA mini-apps, has been openly stored on https://doi.org/10.5281/zenodo.10412696 ^41^ under license: GNU General Public License v3.0. While the subsequent releases will be updated on this Zenodo repository, the history of changes and up to date developments can be checked from our GitLab repository https://gitlab.bsc.es/material-science/nomad-mini-apps-suite. The corresponding checkpoints for all the GW mini-apps are stored at https://doi.org/10.5281/zenodo.10142416 ^43^ under license: Apache License 2.0 The FHI-aims mini-app is published under its mother code’s license, so its access has to be managed through the FHI-aims team at fhi-aims.org.
