# Supplementary material for: The NOMAD mini-apps: A suite of kernels from ab initio electronic structure codes enabling co-design in high-performance computing
Source: Open Res Eur. 2025 Apr 10;4:35. Originally published 2024 Feb 19. [Version 3] doi: 10.12688/openreseurope.16920.3 (PMC11224708; doi:10.12688/openreseurope.16920.3)
Supplement: Peer review history

# Reviewer report: Reviewer response for version 3

**DOI:** 10.21956/openreseurope.21749.r53064

**Published:** 2025-05-19

**Authors:** Sarah Neuwirth

**Affiliations:** 1 Johannes Gutenberg University, Mainz, Germany

Thank you for submitting the revised version of your paper. I appreciate the thoughtful updates. All of my previous questions and concerns have been addressed. I therefore recommend the paper for acceptance.

Is the rationale for developing the new software tool clearly explained?

Yes

Is the description of the software tool technically sound?

Partly

Are sufficient details of the code, methods and analysis (if applicable) provided to allow replication of the software development and its use by others?

Yes

Is sufficient information provided to allow interpretation of the expected output datasets and any results generated using the tool?

No

Reviewer Expertise:

Parallel I/O and storage systems, modular supercomputing, performance modeling and analysis, optimization, reproducible benchmarking, and parallel programming models

I confirm that I have read this submission and believe that I have an appropriate level of expertise to confirm that it is of an acceptable scientific standard.

# Reviewer report: Reviewer response for version 2

**DOI:** 10.21956/openreseurope.19181.r41049

**Published:** 2024-07-04

**Authors:** Ernesto Dufrechou

**Affiliations:** 1 Universidad de la Republica Uruguay, Montevideo, Montevideo Department, Uruguay

I think the article has improved. The rationale behind the selection of the miniapps is explained with more detail, and the experiments provide reasonable evidence that the performance of the miniapps and the full apps is closely related. Therefore, to integrate these miniapps in a co-design strategy for large-scale HPC platforms is reasonable, perhaps alongside other aspects such as BLAS-LAPACK performance or accelerator capabilities that are useful for other applications.

Is the rationale for developing the new software tool clearly explained?

Yes

Is the description of the software tool technically sound?

Partly

Are sufficient details of the code, methods and analysis (if applicable) provided to allow replication of the software development and its use by others?

Yes

Is sufficient information provided to allow interpretation of the expected output datasets and any results generated using the tool?

Partly

Reviewer Expertise:

I research in heterogeneous computing and HPC. My main lines of work revolve around the accelleration of linear algebra kernels and scientific applications using GPUs.

I confirm that I have read this submission and believe that I have an appropriate level of expertise to confirm that it is of an acceptable scientific standard.

# Reviewer report: Reviewer response for version 2

**DOI:** 10.21956/openreseurope.19181.r41048

**Published:** 2024-07-04

**Authors:** Jerzy Proficz

**Affiliations:** 1 Politechnika Gdanska, Gdańsk, Pomeranian Voivodeship, Poland

The proposed improvements are sufficient for accepting the paper.

Is the rationale for developing the new software tool clearly explained?

Yes

Is the description of the software tool technically sound?

Yes

Are sufficient details of the code, methods and analysis (if applicable) provided to allow replication of the software development and its use by others?

Yes

Is sufficient information provided to allow interpretation of the expected output datasets and any results generated using the tool?

Yes

Reviewer Expertise:

HPC, energy efficiency, software/hardware optimization, distributed/parallel applications

I confirm that I have read this submission and believe that I have an appropriate level of expertise to confirm that it is of an acceptable scientific standard.

# Reviewer report: Reviewer response for version 1

**DOI:** 10.21956/openreseurope.18286.r38376

**Published:** 2024-05-03

**Authors:** Sarah Neuwirth

**Affiliations:** 1 Johannes Gutenberg University, Mainz, Germany

This article introduces the NOMAD mini-applications suite, which is based on kernels from ab initio electronic code structures participating in the NOMAD Center of Excellence. The NOMAD mini-apps suite focuses on four flagship applications, i.e., the ELPA eigensolver library and the GW implementations of the exciting, Abinit, and FHI-aims codes. The authors identify the most relevant kernels of those four applications by profiling runs with different configurations on the MareNostrum-4 supercomputer. The objective of the benchmark suite is to facilitate and enable co-design of future High-Performance Computing (HPC) systems with a focus on ab initio computational materials science codes.

 Mini-applications serve as crucial benchmarks in HPC systems due to their ability to simulate specific aspects of real-world applications efficiently. These compact programs encapsulate key computational patterns and performance characteristics, allowing for targeted analysis and optimization. By focusing on critical computational kernels or algorithms, mini-applications provide insights into system behavior, scalability, and bottlenecks, aiding in the evaluation and comparison of HPC architectures and software stacks. Moreover, their simplicity facilitates rapid experimentation and tuning, enabling researchers and engineers to fine-tune system configurations for optimal performance, ultimately advancing the design and deployment of HPC solutions.

 Overall, this article addresses a timely topic and describes the four application kernels well. The article has a clear structure and is mostly well written. Nevertheless, some questions remain unanswered after reading the paper and I would like to encourage the authors to clarify these points in a revision, specifically regarding the co-design aspect and applicability to large-scale systems.

 Comments and suggestions:

 In the introduction, the authors state that “mini-app benchmarking can inform the implementation of new HPC systems more effectively”. Furthermore, the authors say that “Mini-apps have the potential to support our efforts in co-design as the performance metrics will be easily transferable between selected HPC systems and pre/post-exascale prototypes more effectively”. While these statements make sense, there is no further clarification on how the presented mini-apps can benefit a co-design cycle. Also, the authors do not further elaborate about selected or targeted performance metrics, even though they mention the (simplified) Sustained System Performance ((S)SSP). To further emphasize the impact of mini-app suites such as the NOMAD suite, it may be helpful to further explain performance metrics for the broader audience.

 Although the authors provide a good description of their kernel selection process based on the traces, it is not clear how representative these are for the overall performance of the application. For example, although the ELPA mini-app explains why the trans_ev_tridi_to_band subroutine is selected, most of the application's time is spent in DGEMM, so a complete exclusion of the BLAS library seems questionable. Especially since BLAS is a widely used library.

 Furthermore, it is not explained for any of the four applications why the authors chose particular configurations for tracing and to what extent these are representative of the actual applications. I would encourage the authors to further explain their methodology.

 Although most modern scientific applications use GPUs, the four mini-apps presented focus on purely CPU-based codes. Especially with regard to the aforementioned co-design process, it would be beneficial if the NOMAD suite also integrated GPU-accelerated applications.

 Finally, I would like to point out to the authors that it would be helpful for readers to include a small example of the applicability of the NOMAD mini-app suite. This example could be used to briefly explain what insights the mini-apps can provide (e.g., performance metrics and provided output/results) and how these can then be used in the context of co-design.

Is the rationale for developing the new software tool clearly explained?

Yes

Is the description of the software tool technically sound?

Partly

Are sufficient details of the code, methods and analysis (if applicable) provided to allow replication of the software development and its use by others?

Yes

Is sufficient information provided to allow interpretation of the expected output datasets and any results generated using the tool?

No

Reviewer Expertise:

Parallel file and storage systems, reproducible benchmarking, holistic performance engineering (i.e., system monitoring, performance modeling, performance optimization), modular supercomputing (i.e., resource disaggregation and virtualization), high performance computing and networking, parallel I/O, and parallel programming models

I confirm that I have read this submission and believe that I have an appropriate level of expertise to state that I do not consider it to be of an acceptable scientific standard, for reasons outlined above.

# Response

**Published:** 2024-05-07

**Authors:** Julio Gutierrez Moreno

**Affiliations:** Barcelona Supercomputing Center, Barcelona, Cadiz, Spain

This article introduces the NOMAD mini-applications suite, which is based on kernels from ab initio electronic code structures participating in the NOMAD Center of Excellence. The NOMAD mini-apps suite focuses on four flagship applications, i.e., the ELPA eigensolver library and the GW implementations of the exciting, Abinit, and FHI-aims codes. The authors identify the most relevant kernels of those four applications by profiling runs with different configurations on the MareNostrum-4 supercomputer. The objective of the benchmark suite is to facilitate and enable co-design of future High-Performance Computing (HPC) systems with a focus on ab initio computational materials science codes. Mini-applications serve as crucial benchmarks in HPC systems due to their ability to simulate specific aspects of real-world applications efficiently. These compact programs encapsulate key computational patterns and performance characteristics, allowing for targeted analysis and optimization. By focusing on critical computational kernels or algorithms, mini-applications provide insights into system behavior, scalability, and bottlenecks, aiding in the evaluation and comparison of HPC architectures and software stacks. Moreover, their simplicity facilitates rapid experimentation and tuning, enabling researchers and engineers to fine-tune system configurations for optimal performance, ultimately advancing the design and deployment of HPC solutions. Overall, this article addresses a timely topic and describes the four application kernels well. The article has a clear structure and is mostly well written. Nevertheless, some questions remain unanswered after reading the paper and I would like to encourage the authors to clarify these points in a revision, specifically regarding the co-design aspect and applicability to large-scale systems.  

**Comments and suggestions:** **Comment #1:** In the introduction, the authors state that “mini-app benchmarking can inform the implementation of new HPC systems more effectively”. Furthermore, the authors say that “Mini-apps have the potential to support our efforts in co-design as the performance metrics will be easily transferable between selected HPC systems and pre/post-exascale prototypes more effectively”. While these statements make sense, there is no further clarification on how the presented mini-apps can benefit a co-design cycle. Also, the authors do not further elaborate about selected or targeted performance metrics, even though they mention the (simplified) Sustained System Performance ((S)SSP). To further emphasize the impact of mini-app suites such as the NOMAD suite, it may be helpful to further explain performance metrics for the broader audience.

**Reply: **We thank the reviewer for her insightful feedback on our paper. We appreciate the comments regarding the impact of our mini-app suites in the co-design process and agree with the need for further clarification in some of the sections. Some examples of how these mini-apps are currently used for co-design are now given in the conclusions section and highlighted in the updated Figure 1. These include the continuous benchmarking of several peta- and pre-exascale architectures in (pre)operational stages and the porting to prototype hardware that will be potentially used in future exa- and post-exascale platforms. Our mini-apps could also be helpful as a starting point for implementing new porting strategies and optimisations before merging them with the full code. The codes included in the suite use different ab initio approaches, typically characterised by different bottlenecks. The new release of the mini-apps, published along with the revised version of the manuscript, contains benchmark scripts that launch multiple executions, progressively increasing the number of independent (single-core or threaded) processes until they fill all the CPUs in a node. These scripts offer options for compilation and execution on MareNostrum-5, MareNostrum-4, and CTE-POWER (BSC, Spain), Leonardo (CINECA, Italy), LUMI (CSC, Finland), Vega (IZUM, Slovenia) and Karolina (IT4I, Czech Republic). The user can compare how the different mini-apps perform on different machines, analyse the potential degradation of the single-core performance with the occupancy of the node, or test different compiling options simply by looking at the timestamps that are printed as outputs for all the different setups. This upgrade has been commented on in the operation section.  

**Comment #2:** Although the authors provide a good description of their kernel selection process based on the traces, it is not clear how representative these are for the overall performance of the application. For example, although the ELPA mini-app explains why the trans_ev_tridi_to_band subroutine is selected, most of the application's time is spent in DGEMM, so a complete exclusion of the BLAS library seems questionable. Especially since BLAS is a widely used library.

**Reply:** We thank these comments on the mini-app selection, which are very much in line with some of the questions raised by reviewer #1. We agree that our performance analyses could have needed to have been sufficiently explained in some of the cases. In the specific case of ELPA, the original version showed routines at a lower level than the ones that were presented in the manuscript. We understand that the relevance of the routines included in the mini-app could not be straightforwardly understood and, therefore, tridi_to_band (abbreviation for trans_ev_tridi_to_band) was represented mainly by a child subroutine called compute_hh_trafo. In the new version, the figure has been updated, and the trans_ev_tridi_to_band routine is shown without the compute_hh_trafo child subroutine, which allows for a clear distinction of the relevance of this subroutine within the mini-app. This routine takes 32% of the total time in the small test case (8K-element squared matrix) and 39% in the large (100K) one. In addition to the computational time, more features were pointed to in selecting this mini-app, such as the independence of external functions and the effort the ELPA developers made to adapt this kernel to use efficiently vectorial instruction on different hardware. While measuring the performance of linear algebra functions, such as the ones in BLAS, is attractive for benchmarking purposes, the room for co-design is much more limited than with our selection of kernels.  

**Comment #3: **Furthermore, it is not explained for any of the four applications why the authors chose particular configurations for tracing and to what extent these are representative of the actual applications. I would encourage the authors to further explain their methodology. **Reply: **All our test cases represent realistic executions of technologically relevant materials, and they have been decided upon and discussed with the developers of each of the codes. Based on that, for all the applications, we selected a common case performed on a ZrO _2_ unit cell and a more complex one, which required more computational resources and was used to compare the relative weight of each routine in different situations. For exciting, we used examples with reduced and all-bands scenarios. For Abinit, we used a small ZrO _2_ and a more extensive Zr _2_Y _2_O _7_ system. Last, for FHI-aims, we found that ZrO _2_ was already quite heavy computationally, so we also sampled pure Si to perform our comparison. Although all these tests were already made in the past, they were not extensively described in the initial manuscript, so the updated version has been accordingly revised in these lines.  

**Comment #4: **Although most modern scientific applications use GPUs, the four mini-apps presented focus on purely CPU-based codes. Especially with regard to the aforementioned co-design process, it would be beneficial if the NOMAD suite also integrated GPU-accelerated applications.

**Reply:** We completely understand this view, which was also commented by the other referees. As noted here, most of the largest HPC platforms nowadays are in fact accelerated by GPUs. Several of the ab initio codes represented by these mini-apps have offloaded to GPUs selected parts of their DFT sections, however, these new implementations still need to tackle the GW implementations. We believe that with the increasing computational power that will come along with the arrival of the exascale era, the computationally demanding beyond-DFT methods such as GW will increase their accessibility and popularity among the community, and this is the main reason that drove us to choose GW for the majority of the selection of these mini-apps. It is also important to point out that in addition to the current predominance of GPUs, the future of HPC will also increase in hardware heterogeneity (some of them based on CPUs, which could, for example, operate larger vectors than AVX512, e.g. RISC-V VPUs) and new programming models will arise. In addition to the mentioned benchmarks, our mini-apps could also be helpful as a starting point for implementing new porting strategies and optimisations before merging them with the complete code. A mention of this future scenario has been added to the conclusions.

**Comment #5: **Finally, I would like to point out to the authors that it would be helpful for readers to include a small example of the applicability of the NOMAD mini-app suite. This example could be used to briefly explain what insights the mini-apps can provide (e.g., performance metrics and provided output/results) and how these can then be used in the context of co-design.

**Reply:** We thank the referee again for her comments. The NOMAD mini-apps suite is being used to continuously benchmark some of the supercomputers that are being currently deployed by the European High-Performance Computing Joint Undertaking (EuroHPC JU), all having different hardware and compilers/software stacks. Moreover, we have started activities on porting and testing these mini-apps to prototype architectures that will be used in future systems. The outcomes of these activities provide useful feedback to integrators, system administrators, that will eventually benefit all users. The tests carried out on experimental platforms are only feasible with mini-apps, and they are clear examples of co-design, in which the software is adapted to perform efficiently in these novel architectures while we provide constant feedback to the hardware architects and compiler developers at the early stage of development. Comments on these lines have also been added to the conclusions section of the manuscript. We should also mention that along with the updated version of the manuscript, we have also released the new 1.1 version of the suite, accessible at the same repository. In this version, we provide submission scripts to run our benchmarks on several EuroHPC machines: MareNostrum-4, MareNostrum-5, CTE-Power, Leonardo, LUMI, Karolina and Vega. Therefore, the execution of the suite would be straightforward to a user with access to any of these machines. Indications on this have been added to the operations section.

# Reviewer report: Reviewer response for version 1

**DOI:** 10.21956/openreseurope.18286.r38382

**Published:** 2024-04-24

**Authors:** Jerzy Proficz

**Affiliations:** 1 Politechnika Gdanska, Gdańsk, Pomeranian Voivodeship, Poland

The NOMAD mini-apps consist of a small collection of benchmarks focusing on ab initio electronic calculations, intended for a broad range of HPC systems. The concept of co-design is outlined, alongside examples of typical usage scenarios. The fundamental theory underlying the solutions is discussed. While I find the software interesting, I believe further elaboration on the following points would enhance its utility:

1. It would be beneficial to include a figure or table delineating the flow from typical usage/problem description to the specific mini-applications.
2. I am eager to observe results from running the benchmarks on an actual HPC system, particularly a comparative analysis between CPS and GPU architectures.

Overall, the NOMAD mini-apps paper presents a promising approach to ab initio electronic calculations, offering a comprehensive overview of co-design principles and typical usage scenarios, while also demonstrating potential for further enhancement through concrete benchmarking on real HPC systems.

Is the rationale for developing the new software tool clearly explained?

Yes

Is the description of the software tool technically sound?

Yes

Are sufficient details of the code, methods and analysis (if applicable) provided to allow replication of the software development and its use by others?

Yes

Is sufficient information provided to allow interpretation of the expected output datasets and any results generated using the tool?

Yes

Reviewer Expertise:

HPC, energy efficiency, software/hardware optimization, distributed/parallel applications

I confirm that I have read this submission and believe that I have an appropriate level of expertise to confirm that it is of an acceptable scientific standard, however I have significant reservations, as outlined above.

# Response

**Published:** 2024-04-26

**Authors:** Julio Gutierrez Moreno

**Affiliations:** Barcelona Supercomputing Center, Barcelona, Cadiz, Spain

We would like to thank the journal’s editorial team and reviewers for their time, comments, and suggestions, which we believe have helped us improve our manuscript. The revision was made according to the referee’s requirements, and we answered all points made. Please see the responses below.  

**Comment #1:** The NOMAD mini-apps consist of a small collection of benchmarks focusing on ab initio electronic calculations, intended for a broad range of HPC systems. The concept of co-design is outlined, alongside examples of typical usage scenarios. The fundamental theory underlying the solutions is discussed.

**Reply: **We thank the comments provided by the referee and his remarks on the relevance of our mini-apps as a benchmark tool and co-design vehicle for HPC.  

**Comment #2: **While I find the software interesting, I believe further elaboration on the following points would enhance its utility: It would be beneficial to include a figure or table delineating the flow from typical usage/problem description to the specific mini-applications. **Reply: **We thank the reviewer for pointing out the aspects of the potential usage of these mini-apps, which may need to be explained in more detail. All our test cases represent realistic executions of technologically relevant materials, and they have been decided upon and discussed with the developers of each of the codes. In addition, the codes included in the suite use different approaches and basis sets, including a wide range of the most widely used ab initio methods (plane waves and localised orbitals, with all-electron and pseudopotentials) and represent different bottlenecks. These include continuously benchmarking several pre-exascale architectures in (pre)operational stages and porting to prototype hardware potentially used in future (post)-exascale platforms. Moreover, our mini-apps could also be helpful as a starting point for implementing new porting strategies and optimisations before merging them with the complete code. These aspects are now mentioned in the conclusions of the updated manuscript. Also, Figure 1 and its caption have also been updated to comment on these concrete usages.  

**Comment #3: **I am eager to observe results from running the benchmarks on an actual HPC system, particularly a comparative analysis between CPS and GPU architectures.

**Reply: **We understand the point raised here by the referee. As he noted, most of the largest HPC platforms nowadays are accelerated by GPUs. However, we should mention that while some of the ab initio codes represented by these mini-apps have some portions ported to GPUs, these new implementations still need to tackle the GW parts. More details on the codes and computing methodology used in the suite are given in our previous response, comment #5 from referee #1. The NOMAD suite is being used to continuously benchmark some of the supercomputers that are being currently deployed by the European High-Performance Computing Joint Undertaking (EuroHPC JU) and prototype architectures that will be used in future systems, all having different hardware and compilers/software stacks. While the outcomes of these activities provide useful feedback to integrators, system administrators, and users, some of these machines are in the preliminary development stages, and some of the benchmarks we have carried out cannot be publicly disclosed. Also, considering that many of these HPC systems are still being fine-tuned, the outcomes from the benchmarks can vary depending on the exact time the picture is taken. Nevertheless, all these are clear examples of co-design, in which the software is adapted to perform efficiently in these novel architectures while we provide constant feedback to the hardware architects and compiler developers at the early stage of development. Comments on these lines have also been added to the conclusions section of the manuscript. Along with the updated version of the manuscript, we have also released the new 1.1 version of the suite, accessible at the same repository. In this version, we provide submission scripts to run our benchmarks on several EuroHPC machines: MareNostrum-4, MareNostrum-5, CTE-Power, Leonardo, LUMI, Karolina and Vega. Therefore, for a user with access to any of these machines, the compilation (with different compilers) and the execution of the suite should be straightforward. Of course, these scripts can also be adapted to new environments. Indications on this have been added to the operations section.  

**Comment #4:** Overall, the NOMAD mini-apps paper presents a promising approach to ab initio electronic calculations, offering a comprehensive overview of co-design principles and typical usage scenarios, while also demonstrating potential for further enhancement through concrete benchmarking on real HPC systems.

**Reply: **We thank you again for the comments and further suggestions for improving our manuscript.

# Reviewer report: Reviewer response for version 1

**DOI:** 10.21956/openreseurope.18286.r38384

**Published:** 2024-03-18

**Authors:** Ernesto Dufrechou

**Affiliations:** 1 Universidad de la Republica Uruguay, Montevideo, Montevideo Department, Uruguay

The article discusses a software tool designed to assess the performance of High-Performance Computing (HPC) platforms. This tool utilizes small mini-apps to capture the most characteristic computations of larger scientific software applications, particularly excerpts from applications of ab initio quantum chemistry methods. The purpose of this tool is to enable HPC hardware designers to quickly estimate the performance of larger applications on their designs by running lightweight mini-apps.

 Molecular dynamics problems and quantum chemistry are critical applications that motivate the development of HPC hardware capable of running these applications efficiently. However, utilizing large simulations to evaluate a prototype hardware platform can be time and resource-consuming, making it impractical. The mini-app suite is designed to address this issue.

 While the description of the software tool is clear, there is room for improvement in certain areas. The four mini-apps that form the main components of the tool contain selected kernels of the respective larger applications. In some cases, it may be difficult to match the mini-app kernel with the traces. For instance, the ELPA mini-app's chosen kernel is trans_ev_tridi_to_band, but the trace reveals the function tridi_to_band that accounts for only 4% of the execution time. Furthermore, the trace's different green colors make it challenging to identify the portions of the trace corresponding to that function. Although the authors provide arguments for this choice, it remains unclear whether this function's performance characterizes the performance of the entire app. Therefore, choosing a set of these functions may be more appropriate. Additionally, the BLAS (Basic Linear Algebra Subprograms) performance is critical in ELPA's case.

 In general, the article would benefit from a deeper explanation of how the performance of the mini-apps relates to the performance of the larger applications, and under what assumptions the suite can be used in a co-design workflow.

 Another area that requires further clarification is the criteria for the trace configuration. Parameters such as the block size and the number of threads and processes are provided, but their explanations are absent. Moreover, the relative weight of each function may vary depending on the problem size. Unfortunately, Abinit and FHI-aims were tested for only one problem size (35 q-points for Abinit and omitted the case of FHI-aims), while the others were tested for two problem sizes. Conducting systematic tests with more problem sizes and processes/threads configurations (a scaling analysis) could lead to an estimation of the apps' scaling on a new system using the mini-apps.

 Finally, the mini-app suite could benefit from the inclusion of GPU-accelerated codes. This technology is ubiquitous in modern supercomputers and could enhance the tool's versatility and applicability.

Is the rationale for developing the new software tool clearly explained?

Yes

Is the description of the software tool technically sound?

Partly

Are sufficient details of the code, methods and analysis (if applicable) provided to allow replication of the software development and its use by others?

Yes

Is sufficient information provided to allow interpretation of the expected output datasets and any results generated using the tool?

Partly

Reviewer Expertise:

I research in heterogeneous computing and HPC. My main lines of work revolve around the accelleration of linear algebra kernels and scientific applications using GPUs.

I confirm that I have read this submission and believe that I have an appropriate level of expertise to state that I do not consider it to be of an acceptable scientific standard, for reasons outlined above.

# Response

**Published:** 2024-04-11

**Authors:** Julio Gutierrez Moreno

**Affiliations:** Barcelona Supercomputing Center, Barcelona, Cadiz, Spain

We would like to thank the journal’s editorial team and reviewer for his time, comments and suggestions, which we believe have helped me to improve our manuscript. The revision was made according to the referee’s requirements and we answered (in black) all points made. Please see the responses below.  

**Comment** **#1:** The article discusses a software tool designed to assess the performance of High-Performance Computing (HPC) platforms. This tool utilizes small mini-apps to capture the most characteristic computations of larger scientific software applications, particularly excerpts from applications of ab initio quantum chemistry methods. The purpose of this tool is to enable HPC hardware designers to quickly estimate the performance of larger applications on their designs by running lightweight mini-apps. Molecular dynamics problems and quantum chemistry are critical applications that motivate the development of HPC hardware capable of running these applications efficiently. However, utilizing large simulations to evaluate a prototype hardware platform can be time and resource-consuming, making it impractical. The mini-app suite is designed to address this issue.

**Reply** **:** We thank the reviewer for the suggestions for improving our manuscript. We appreciate the recognition of the relevance of ab initio electronics structure calculations in HPC and the importance of having mini-apps to test these codes on prototype hardware platforms.  

**Comment** **#2:** While the description of the software tool is clear, there is room for improvement in certain areas. The four mini-apps that form the main components of the tool contain selected kernels of the respective larger applications. In some cases, it may be difficult to match the mini-app kernel with the traces. For instance, the ELPA mini-app's chosen kernel is trans_ev_tridi_to_band, but the trace reveals the function tridi_to_band that accounts for only 4% of the execution time. Furthermore, the trace's different green colors make it challenging to identify the portions of the trace corresponding to that function. Although the authors provide arguments for this choice, it remains unclear whether this function's performance characterizes the performance of the entire app. Therefore, choosing a set of these functions may be more appropriate. Additionally, the BLAS (Basic Linear Algebra Subprograms) performance is critical in ELPA's case.

**Reply** **:** We thank the reviewers for the comments provided, and we agree that our performance analyses could have needed to have been sufficiently explained in some of the cases. In the specific case of ELPA, the figure in the previous version showed routines at a lower level than the ones that were presented in the manuscript. We understand that the relevance of the routines included in the mini-app could not be straightforwardly understood and, therefore, *tridi_to_band (abbreviation for trans_ev_tridi_to_band) *was represented mainly by a child subroutine called compute_hh_trafo. In the new version, the figure has been updated and the *trans_ev_tridi_to_band* routine is shown without *compute_hh_trafo* child subroutine, which allows for a clear distinction of the relevance of this subroutine within the mini-app. This routine takes 32% of the total time in the small test case and 39% in the large one. In addition to the computational time, more features were pointed to in selecting this mini-app, such as the independence of external functions and the effort the ELPA developers made to adapt this kernel to use vectorial instruction on different hardware efficiently. While measuring the performance of linear algebra functions, such as the ones in BLAS, is attractive for benchmarking purposes, the room for co-design is much more limited than with our selection of kernels. We agree that the colour-coding use in the traces could have been clearer; therefore, we have updated the colours in Figures 3 and 4.  

**Comment** **#3:** In general, the article would benefit from a deeper explanation of how the performance of the mini-apps relates to the performance of the larger applications, and under what assumptions the suite can be used in a co-design workflow.

**Reply: **The manuscript has been revised with a description of the test cases used for each code. Moreover, a short description of their performance compared to the test case selected for the mini-app has been included. More details are given in the next comment. The manuscript has been accordingly revised to include this. Some examples of how these mini-apps are currently used for co-design are also given in the conclusions section. These include the continuous benchmarking of several peta- and pre-exascale architectures in (pre)operational stages and the porting to prototype hardware that will be potentially used in future exa- and post-exascale platforms. Comments mentioning these uses have been added to the conclusions section.  

**Comment** **#4:** Another area that requires further clarification is the criteria for the trace configuration. Parameters such as the block size and the number of threads and processes are provided, but their explanations are absent. Moreover, the relative weight of each function may vary depending on the problem size. Unfortunately, Abinit and FHI-aims were tested for only one problem size (35 q-points for Abinit and omitted the case of FHI-aims), while the others were tested for two problem sizes. Conducting systematic tests with more problem sizes and processes/threads configurations (a scaling analysis) could lead to an estimation of the apps' scaling on a new system using the mini-apps.

**Reply: **We should clarify that we have performed exhaustive performance analyses using different test cases for each of the applications and performed scaling tests on the number of MPI/OMP processes to accommodate the available memory resources within a reasonable time to solution. All our test cases represent realistic executions of technologically relevant materials, and they have been decided upon and discussed with the developers of each of the codes. Based on that, for all the applications, we selected a common case performed on a ZrO _2_ unit cell and a more complex one, which required more computational resources and was used to compare the relative weight of each routine in different situations. More concretely, in the case of ELPA, we used squared matrices with sizes of 8K and 100K. For exciting, we used examples with reduced and all bands scenarios. For Abinit, we used a small ZrO _2_ and a more extensive Zr _2_Y _2_O _7_ system. Last, for FHI-aims, we found that ZrO _2_ was already quite heavy computationally, so we also sampled pure Si to perform our comparison. Although all these tests were already made in the past, they were not extensively described in the initial manuscript, so the updated version has been accordingly revised in these lines.  

**Comment** **#5:** Finally, the mini-app suite could benefit from the inclusion of GPU-accelerated codes. This technology is ubiquitous in modern supercomputers and could enhance the tool's versatility and applicability.

**Reply** **:** We understand the point raised here by the referee. As he noted, most of the largest HPC platforms nowadays are accelerated by GPUs. However, we should mention that, while some of the ab initio codes represented by these mini-apps have some portions ported to GPUs, these new implementations still need to tackle the GW parts. Here, we should recall that DFT has been the main workhorse of the ab initio electronic structure community; therefore, it is understandable that developers prioritise the porting of the DFT sections of the code to GPUs. However, the accuracy of DFT in estimating properties such as the band-gap may need to be improved for some applications, and more accurate (and computationally expensive) beyond-DFT methods such as GW are required. We strongly believe that with the increasing computational power that will come along with the arrival of the exascale era, these methods will increasingly become more accessible and popular among the community, and this is the main reason that drove us to choose GW for the majority of the selection of these mini-apps. It is also important to point out that in addition to the current predominance of GPUs, the future of HPC will also increase in hardware heterogeneity (some of them based on CPUs, which could operate larger vectors, e.g. VPUs) and programming models. Our mini-apps could also be helpful as a starting point for implementing new porting strategies and optimisations before merging them with the complete code. A mention of this future scenario, including part of the text in this response, has also been added to the conclusions.
